# Navigating preanalytical challenges: a real-world study on single-tube pneumatic tube systems

**DOI:** 10.3389/fphys.2026.1700252

**Published:** 2026-02-10

**Authors:** Lisha Li, Xiaofei Zhang, Nannan Xu, Miaoli Shao, Binxuan Chen, Feng Wu, Qi Zhao, Wensheng Wang, Jianxin Yan, Zhiheng Wang, Renfang Zhou

**Affiliations:** 1 Department of Clinical Laboratory, The First People’s Hospital of Wenling, Affiliated Wenling Hospital, Wenzhou Medical University, Taizhou, China; 2 College of Mechanical Engineering, Zhejiang University of Technology, Hangzhou, China

**Keywords:** blood specimen collection, clinical chemistry, hemolysis, laboratories, pneumatic transportation system

## Abstract

**Purpose:**

Pneumatic tube systems (PTS) accelerate the transport of samples in the laboratory but may lead to preanalytical hemolysis. Samples may rest before transport during busy times, but the effect on test accuracy is unclear. This study presents a novel single-tube PTS with a secondary deceleration device and LIS integration, evaluating its impact on routine biochemical parameters and examining the effects of prolonged sample resting times before PTS transport.

**Methods:**

In a prospective study of 34 outpatients, four blood samples per participant were collected in silica-clot activator tubes. Samples were randomly allocated to: immediate PTS transport (Group A), PTS after 15-min rest (B), PTS after 30-min rest (C), or manual delivery (Group D). All samples from each subject were centrifuged and analyzed simultaneously upon arrival. Twenty-two biochemical parameters were measured; results for PTS groups were compared to the manual control, with bias assessed against ±1/2 total allowable error (±1/2TEa).

**Results:**

The hemolysis index (HI) was negative for both PTS and HD groups. Lactate dehydrogenase (LDH) levels were significantly elevated in all PTS groups versus manual delivery (*p* < 0.001). The bias for LDH was clinically acceptable only for immediate transport (Group A: +3.2%) but exceeded limits after 15- and 30-min rests (Group B: +5.8%; Group C: +10.7%). Glucose showed significant yet clinically acceptable variations. Other parameters remained within acceptable limits.

**Conclusion:**

The single-tube PTS is suitable for transporting blood specimens for biochemical analysis. To maintain the reliability of biochemical results from silica-clot activator tubes, laboratories should minimize pre-transport delays and include resting time as a critical variable in PTS validation protocols.

## 1 Introduction

Efficient specimen transport is vital for quality and efficiency in clinical labs, with systems like pneumatic tube systems (PTS) designed to quickly transport specimens and reduce turnaround time. Traditional PTS operates by using air pressure to move items through bullet-shaped containers. A single pathway can handle only one transport task at a time, limiting its capacity for large specimen transfers. Currently, the majority of specimens requiring transportation in clinical laboratories are blood samples contained in standard type of vacuum tubes. A novel single-tube PTS, which facilitates the continuous conveyance of samples at a constant velocity of approximately 10 m per second without the use of containers, is increasingly gaining popularity. It integrates with the laboratory information system for an automated, “closed-loop” specimen reception and sorting process. This integration improves specimen transmission efficiency and accuracy in clinical labs (Yu et al., 2024). While single-tube PTS offers advantages over traditional PTS, it differs significantly in speed, path, and dynamic forces. Individual tubes are subjected to more intense shocks (Luginbühl et al., 2024), which can lead to the damage of blood cells, resulting in hemolysis and significant alterations in biochemical results. These changes can substantially affect clinical decisions and outcomes. Current research has examined how PTS affects blood gases, hematology, clinical chemistry, coagulation, thromboelastometry, urine samples, and platelet function (Andersen and Brandslund, 2021; Nybo et al., 2018; Slavík et al., 2020). Meta-analyses (Kapoula et al., 2017) suggest PTS can cause hemolysis, raising lactate dehydrogenase (LDH), aspartate aminotransferase (AST), and potassium ions (K^+^) levels, though effects vary by study. Factors like transport rate, distance, accelerations, and area under the curve were reported to influence lab results (Kapoula et al., 2017; Stangerup et al., 2021; Huang et al., 2025). Consequently, it is crucial to assess its impact on biochemical parameters, particularly those sensitive to hemolysis, prior to the implementation of a single-tube PTS.

In practical applications, it has been observed that certain specimens experience a resting period between their collection and subsequent transportation via the PTS. This issue is most evident during peak blood collection times when the number of specimens surpasses the PTS transport capacity, causing some samples to wait for dispatch. In some laboratories, there is no direct connection between the blood collection line and the single-tube PTS, which results in a certain amount of resting time before the sample is transferred. This resting is of particular concern due to its potential impact on pre-analytical errors. Previous studies have demonstrated a significant association between the pre-transport condition of blood specimens and preanalytical hemolysis. For instance, (Yang et al., 2023) found that centrifuging or isolating serum prior to PTS transport reduces bias in LDH results. Similarly, (Luginbühl et al., 2024) reported an increase in HI when comparing the transportation of half-filled tubes to fully-filled tubes. To date, no research has examined the effects of pre-transport samples resting on laboratory test outcomes when using a single-tube PTS.

Consequently, this study aims to address two primary objectives. The first objective is to evaluate the impact of a novel single-tube PTS, which includes a secondary deceleration mechanism, on twenty-two biochemical parameters. The second objective is to determine whether the duration of the sample resting prior to PTS transport affects conventional biochemical parameters. Samples transported via the PTS were classified according to different standstill durations and compared with those transported manually, with the differences between the two groups being analyzed.

## 2 Materials and methods

### 2.1 Subjects

Blood samples for this study were collected from 34 outpatients (10 males and 24 females) at the First People’s Hospital of Wenling, aged 18 to 60, with an average age of 39.67 ± 12.92 years. The study was approved by the Ethics Committee of the First People’s Hospital of Wenling (KY-2024-1001-01). Written consent was obtained before the individual blood collection.

### 2.2 Instruments, reagents, and consumables

The system utilized in this experiment was a fully automated single-tube pneumatic transport system (Model: ELA1600AMD), designed and supplied by Hangzhou Sino Technology Co., Ltd. The single-tube pneumatic transport system comprises a sending unit, transmission pipeline, secondary deceleration device, and receiving unit. The complete transport path in the PTS is as follows: The specimen enters the sending unit, where compressed air is used to push the specimen into the transmission pipeline. At the end of the pipeline, a secondary deceleration device (featuring a first-stage reverse airflow deceleration and second-stage high-soft-brush active energy-absorbing deceleration) gently reduces the high-speed flow of the specimen until it reaches the receiving unit. The primary function of the receiving unit is to confirm the arrival of the specimen.

**FIGURE 1:**
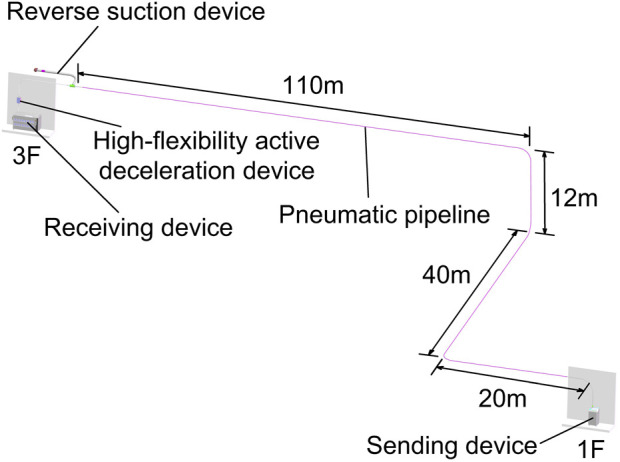
Layout of the fully automated single-tube pneumatic tube system at the First People’s Hospital of Wenling.

The experimental setup was arranged as follows: the sending unit was positioned on the first floor in the clinical specimen processing area, while the receiving unit was located on the third floor in the clinical specimen processing center. The pipeline route was designed according to the site conditions, with the specimen traveling a distance of 182 m through the pipeline (Figure 1). The initial sending pressure was set to 0.25 MPa, and the relay pressure was adjusted to 0.28 MPa. The initial pressure launched the tube vertically upwards at a gradual speed (ranging from 0 to 10 m/s), while the relay pressure maintained the tube’s speed for inter-floor transport (approximately 10 m/s). The operating temperature of the equipment was maintained under controlled room temperature conditions (18 °C–25 °C).

The Beckman Coulter AU5800 analyzer (Brea, CA, United States) was used to detect routine chemistry tests. The tests included albumin, alkaline phosphatase, alanine aminotransferase, AST, total calcium, creatine kinase, creatinine, direct bilirubin, gamma-glutamyltransferase, glucose, 2-hydroxybutyrate dehydrogenase, inorganic phosphorus, LDH, magnesium, total bilirubin, total protein, urea, uric acid, sodium, K^+^ and chloride. The HI was also assessed using the reagent supplied by Beckman Coulter (Brea, United States), with results reported semi-quantitatively as N, +, ++, and +++. The “N” designation indicates hemoglobin concentrations below 50 mg/dL. The calibrators and reagents used in the experiment were provided by the manufacturer of the analyzer. Third-party quality control materials were supplied by Bio-Rad Laboratories.

### 2.3 Specimen collection, transport, and testing

**FIGURE 2:**
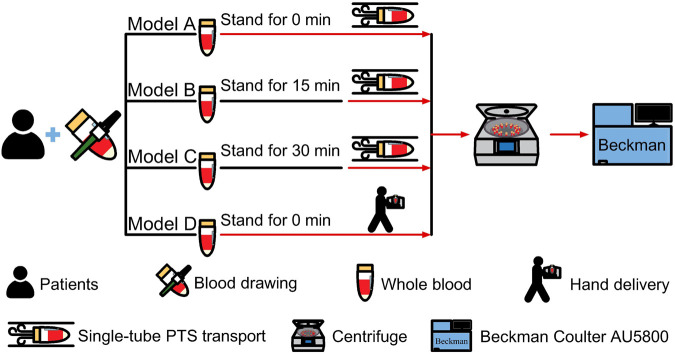
Blood sample processing workflow. Following collection from patients, samples were subjected to varying standing times (Model A: 0 min; B: 15 min; C: 30 min; D: 0 min) prior to transport. Models A–C utilized a single-tube pneumatic tube system (PTS), while Model D was hand-delivered (HD). All samples were subsequently centrifuged and analyzed on a Beckman Coulter AU5800. Icons depict key procedural steps.

The phlebotomy was performed by the same trained nurse using a “BD Vacutainer Flashback 21 GA*1” (0.8 mm*25 mm) needle from the anterior elbow vein of the enrolled patient in accordance with the standardized operating procedures for specimen collection.Blood was collected in a 5.0 mL SST vacuum blood collection tube with a yellow cap (product number 367986, batch number 3257991, Becton Dickinson, Franklin Lakes, NJ, United States) and filled to the specified level. Each participant provided four fasting venous blood samples simultaneously and was assigned to one of four groups: Group A (immediate single-tube PTS transmission), Group B (single-tube PTS transmission after 15 min of static time), Group C (single-tube PTS transmission after 30 min of static time), and Group D (hand delivery, HD). The phlebotomist followed the assigned sequence during venipuncture. The order of blood draw for the four tubes (Groups A-D) was randomized using a computer-generated sequence (R software) to prevent order-related bias. After collection, the tubes were gently inverted 5 times for mixing, following the manufacturer’s instructions. The static specimens in PTS groups were placed upright on the test tube rack immediately after blood collection and were not moved until the specified time point, which was monitored by a designated individual. Upon completion of the designated resting time, the specimens were transferred. Specimens in the single-tube PTS groups (A, B, and C) were transported via the same pneumatic station from the first-floor processing office to the third-floor center, oriented in a base-forward and cap-rearward position The blood specimens in the hand delivery team (Group D) were positioned upright on a test tube rack within a biological transport box. These specimens were transported by a trained specimen transporter immediately following collection, following a standardized walking route (Approximately 10 min). The simultaneous centrifugation and manually loaded for analysis of all specimens (Groups A-D) from each subject were performed upon their collective arrival at the processing center, using the standardized protocol detailed in Figure 2 (3,000 rpm, 4 °C, 10 min). Samples with visible jaundice or lipemia were rejected. All specimen testing was completed within 2 h after collection. Timing details are provided in Supplementary Table S1. The experimental procedures were conducted in accordance with standardized operating procedures for instruments and all tests were performed under qualified quality control conditions. All sample testing and transportation were conducted in our laboratory, which is accredited by the National Accredited Board of Laboratories (ISO 15189:2022).

### 2.4 Statistical analysis

Statistical analysis was performed using SPSS version 27.0 (IBM, New York, United States), MATLAB 2022 (MathWorks, Massachusetts, United States), the DeepWise and Beckman Coulter DxAI platform (https://dxonline.deepwise.com). Plotting of image measurements was performed using SolidWorks 2022 (Dassault Systèmes, Massachusetts, United States), and AutoCAD 2023 (Autodesk, California, United States). The Shapiro-Wilk test was used to test the normality of the parameters. For normally distributed data, results are described as the mean ± standard deviation. Non-normally distributed data is expressed as the median (25th quantile-75th quantile). The paired t-test (normal distribution) and Wilcoxon signed-rank test (non-normal distribution) were used to compare the clinical biochemical indexes between groups A, B, C, and D. For parameters with statistical differences between groups, the bias of the results between the PTS transmission group and manual transportation was calculated. The bias calculation formula was as follows: bias = (pneumatic transmission test result - manual transmission test result)/manual transmission test result * 100%. Outliers in the bias values (A–D, B–D, C–D comparisons) were identified using Tukey’s method on pooled, blinded data. Details are provided in Supplementary Tables S2,S3. The acceptable range total was set at less than one-half of the total allowable error (TEa), in accordance with the interlaboratory evaluation standard of the Clinical Laboratory Center of the National Health Commission of China in 2024. The primary endpoint was analytical bias relative to 1/2 TEa for analytes. Statistical significance was considered secondary and exploratory. The data were plotted using a Bland-Altman plot to illustrate a pattern of differences between the four sets of data. A p-value <0.05 is considered statistically significant.

## 3 Results

### 3.1 The impact of four different transport methods on routine biochemical parameters

None of the samples exhibited macroscopic hemolysis, and the results of the LIH index were negative in all groups. There were no statistically significant differences in levels of AST, K^+^, DBili, Urea, CA, Cl^−^, CREA, UA, and TP between PTS samples and HD samples (all p > 0.05). However, the concentrations of LDH, GLU, and HBDH were statistically significant between groups A and D, groups B and D, as well as groups C and D (all p < 0.05) (Table 1).

**TABLE 1:** Comparative analysis of variations in clinical biochemical indices across four distinct modes of transport.

Analyte, unit	PTS			HD	*p-*value		
	Group A	Group B	Group C	Group D	*p*1	*p*2	*p*3
Albumin, g/L	42.2 ± 4.9	42.6 ± 4.7	42.6 ± 4.8	42.5 ± 4.6	0.004	0.848	0.886
Alanine aminotransferase, U/L	15.6 (10.0–22.4)	15.8 (10.6–21.3)	15.8 (8.4–21.7)	16.1 (10.6–23.0)	0.060	0.492	0.027
Aspartate aminotransferase, U/L	18.6 (15.6–23.8)	20.0 (15.8–22.6)	19.0 (14.7–22.3)	19.5 (16.1–23.0)	0.231	0.132	0.337
Chloride, mmol/L	104.8 ± 1.7	104.7 ± 1.6	104.7 ± 1.6	104.7 ± 1.5	0.100	0.561	0.632
Creatinine, μmol/L	53.8 (48.1–67.0)	54.2 (48.2–65.5)	53.5 (48.6–65.0)	53.9 (47.3–66.2)	0.401	0.695	0.568
Gamma-glutamyltransferase, U/L	17.6 (12.1–21.7)	17.4 (12.2–22.3)	18.4 (11.9–24.0)	18.1 (11.9–22.1)	0.431	0.159	0.016
Glu, mmol/L	5.06 (4.52–5.41)	5.00 (4.53–5.40)	4.88 (4.46–5.42)	4.93 (4.46–5.32)	<0.001	<0.001	0.004
LDH, U/L	169 (151–186)	172 (152–193)	178 (162–194)	160 (147–176)	<0.001	<0.001	<0.001
Magnesium, mmol/L	0.81 (0.74–0.88)	0.82 (0.76–0.88)	0.83 (0.76–0.87)	0.83 (0.74–0.88)	0.008	0.802	0.834
Inorganic phosphorous, mmol/L	1.19 ± 0.19	1.18 ± 0.20	1.19 ± 0.21	1.16 ± 0.18	0.022	0.256	0.077
K^+^, mmol/L	4.18 ± 0.26	4.18 ± 0.27	4.23 ± 0.24	4.18 ± 0.30	0.502	0.943	0.317
Sodium, mmol/L	138.6 ± 1.8	138.8 ± 1.7	139.0 ± 1.7	139.0 ± 2.0	<0.001	0.005	0.084
Total bilirubin, μmol/L	13.2 (8.8–15.4)	12.6 (8.6–15.4)	12.7 (8.6–15.5)	12.6 (8.8–15.6)	0.001	0.074	0.080
Total protein, g/L	70.0 ± 6.0	70.0 ± 5.7	69.8 ± 5.8	69.8 ± 5.5	0.289	0.985	0.751
Uric acid, μmol/L	300 ± 73	299 ± 74	302 ± 70	303 ± 73	0.335	0.962	0.785
Direct bilirubin, μmol/L	1.9 (1.3–2.5)	1.9 (1.3–2.4)	1.9 (1.2–2.4)	1.8 (1.2–2.6)	1.000	0.355	0.324
Alkaline phosphatase, U/L	66.7 (56.0–100.7)	67.4 (55.9–104.0)	67.9 (56.5–105.1)	66.9 (54.9–90.7)	0.015	0.052	0.074
Urea, mmol/L	4.31 (2.56–4.94)	4.34 (2.48–4.95)	4.37 (2.57–4.98)	4.25 (2.49–4.96)	0.907	0.637	0.709
Total calcium, mmol/L	2.31 ± 0.11	2.31 ± 0.11	2.31 ± 0.11	2.31 ± 0.11	0.339	0.325	0.304
Creatine kinase, U/L	73 (50–110)	75 (47–108)	78 (60–107)	76 (50–109)	0.243	0.237	0.027
2-Hydroxybutyrate dehydrogenase, U/L	111 (98–126)	112 (99–127)	115 (100–124)	105 (94–113)	<0.001	<0.001	<0.001
HI	N	N	N	N			

*p*1, *p*2, and *p*3 represent the p-values for differences between groups A and D, B and D, as well as C and D, respectively.

HI, results were reported semi-quantitatively as N, +, ++, and +++.

**FIGURE 3:**
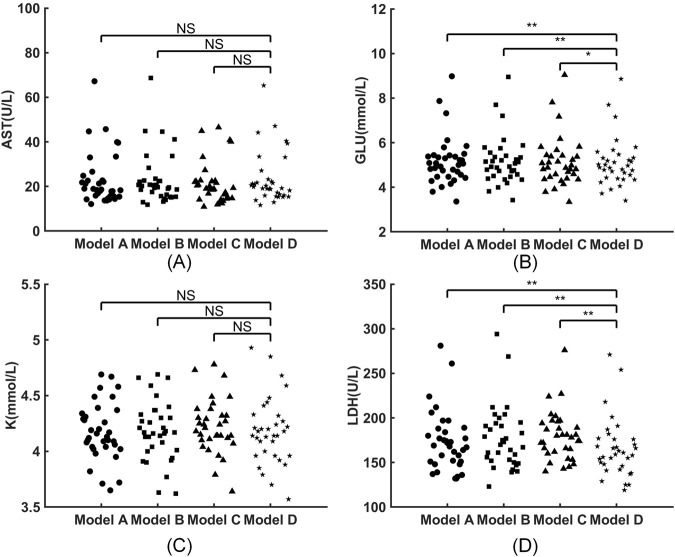
Effects of four different models of sample transportation on routine biochemical tests. **(A)** AST; **(B)** GLU; **(C)** K^+^; **(D)** LDH. NS indicates non-significance (*, *p* < 0.05; **, *p* < 0.001).

There were no statistically significant differences between the four groups in levels of AST and K^+^ (Figures 3A,C). The GLU concentrations in groups A, B, C, and D were 5.06 (4.515–5.405) mmol/L, 5 (4.527–5.397) mmol/L, 4.88 (4.46–5.42) mmol/L, and 4.93 (4.46–5.317) mmol/L, respectively. As the sample was left to rest for a longer period of time before PTS transmission, the GLU concentrations exhibited a gradual decrease (Figure 3B). The concentrations of LDH in Group A, B, C, and D were 169 (151–186) U/L, 171.5 (152.25–193.25) U/L, 178 (162–194) U/L, and 160 (146.5–176) U/L, respectively. Notably, LDH levels in samples of the PTS transmission groups (A, B, and C) were all higher than those of the HD group. As the sample was left to rest for a longer duration before PTS transmission, the LDH concentrations showed a gradual increase (Figure 3D).

### 3.2 Analysis of biases between groups

Biochemical parameters exhibiting significant intergroup differences were identified, and biases were analyzed (A vs. D, B vs. D, C vs. D) to evaluate their adherence to clinical laboratory standards. Except for LDH, the biases for all biochemical parameters between the PTS transmission group and the manual inspection were less than 1/2 TEa, indicating the biases were all acceptable. The LDH bias between groups A and D was acceptable, while the biases between groups B and D, and C and D were not acceptable by established standards (Table 2; Supplementary Table S4).

**TABLE 2:** Examination of biases in indices exhibiting significant differences among four transport modes.

Analyte, unit	Bias(%)			1/2TEa (%)
	A-D	B-D	C-D	
Albumin, g/L	−0.691 ± 1.223	−0.008 ± 1.22	−0.008 ± 1.173	3
Alanine aminotransferase, U/L	−2.953 ± 6.834	0.564 ± 5.083	−2.508 ± 9.621	8
Gamma-glutamyltransferase, U/L	−0.903 ± 3.415	1.685 ± 4.927	2.972 ± 5.759	5.5
Glu, mmol/L	1.248 ± 1.299	1.461 ± 1.589	0.83 ± 1.582	3.5
LDH, U/L	3.207 ± 3.861	5.819 ± 5.057^a^	10.712 ± 6.656^a^	5.5
Magnesium, mmol/L	−0.921 ± 1.901	0.065 ± 1.748	0.078 ± 1.646	7.5
Inorganic phosphorous, mmol/L	0.922 ± 2.788	0.668 ± 2.24	0.859 ± 1.589	5
Sodium, mmol/L	−0.227 ± 0.308	−0.135 ± 0.271	−0.099 ± 0.318	2
Total bilirubin, μmol/L	−1.151 ± 2.219	−0.39 ± 2.11	−0.526 ± 1.875	7.5
Alkaline phosphatase, U/L	1.142 ± 2.662	0.923 ± 2.877	0.732 ± 2.592	9
Creatine kinase, U/L	0.791 ± 3.295	−1.259 ± 3.611	0.452 ± 3.934	7.5
2-Hydroxybutyrate dehydrogenase, U/L	3.446 ± 3.34	6.135 ± 4.632	8.083 ± 4.959	15

TEa, is from the 2024 China National Health Commission Clinical Laboratory Center External Quality Evaluation Standards. Bias <1/2TEa, is within the acceptable range of the laboratory.

^a^ Bias > acceptable range of the laboratory.

**FIGURE 4:**
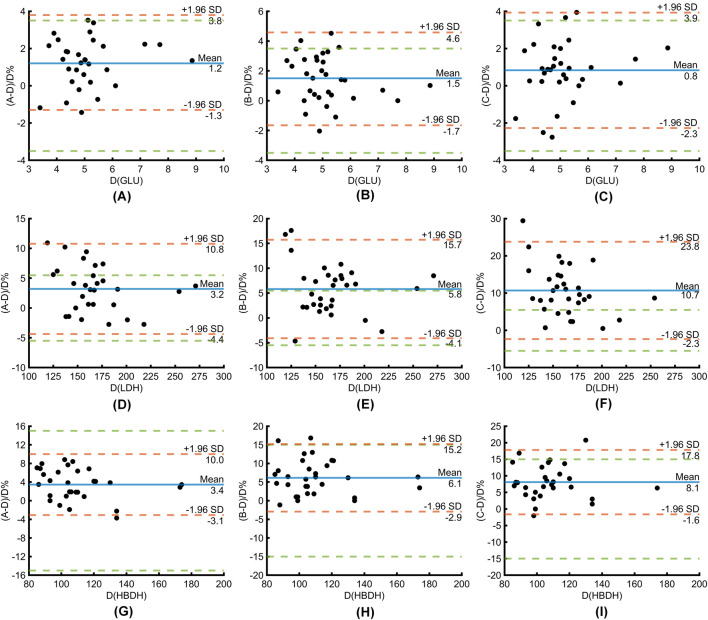
Bland-Altman plots comparing PTS (immediate, 15-, 30-min standing) versus manual delivery for GLU **(A–C)**, LDH **(D–F)** and HBDH **(G–I)**, depicting mean bias (blue line), limits of agreement (red dotted lines, χ±1.96 SD), and allowable bias threshold (green dotted line).

The average biases of GLU results in groups A, B, C, and D were +1.2%, +1.5%, and +0.8%, respectively, all within the acceptable range of the laboratory (Figures 4A–C). In the immediate PTS transmission group (Group A), the average bias of LDH detection was +3.2%, which is below the maximum allowable bias of ±4.4% (Figure 4D). In the 15-min PTS delivery group (Group B), the average bias was +5.8%, exceeding the clinically acceptable limit (Figure 4E). Furthermore, in the 30-min PTS transmission group (Group C), the average bias was +10.7%, which also exceeded the clinically acceptable limit (Figure 4F). The average biases of 2-Hydroxybutyrate dehydrogenase (HBDH) results in groups A, B, C, and D were +3.4%, +6.1%, and +8.1%, respectively, all within the acceptable range of the laboratory (Figures 4G–I).

## 4 Discussion

To our knowledge, this is the first study to validate a single-tube PTS with a secondary deceleration device, and to examine the effects of prolonged pre-transport resting time in this setting. The robustness of the conclusions was confirmed through sensitivity analyses, including multiple comparison correction (Holm procedure) and repeated-measures modeling (Supplementary Table S5). We conducted a comparative analysis of 22 biochemical indicators between specimens transported using single-tube PTS and those delivered manually within clinical laboratory settings. The findings indicated that the biases observed were within acceptable limits. Therefore, this single-tube PTS system is deemed suitable for the transportation of blood specimens for these tests. Based on visual inspection and the negative results of the HI, it was concluded that none of the samples exhibited macroscopic hemolysis in our experiment. In examining other markers of hemolysis, such as K+, AST, and LDH, no statistically significant differences were observed in the levels of K+ and AST between PTS and HD groups. However, significantly elevated LDH levels were detected. This finding may be attributed to the high intracellular concentration of LDH, where even minor cellular rupture can result in a substantial increase in LDH levels. This outcome aligns with the majority of existing studies (Andersen et al., 2017; Lee et al., 2017), but not with Suchsland et al. (2017). Different PTS configurations can induce varying degrees of hemolysis. Discrepancies may arise from variations in PTS operational parameters, such as distance, speed, acceleration, and the inclusion of buffer devices (Ding et al., 2021; Farnsworth et al., 2019; Gils et al., 2020; Cakirca and Erdal, 2017). Luginbühl et al. (2024) recorded the acceleration profiles of two PTS and demonstrated that blood samples transported via a single-tube PTS experience elevated amount of shocks (6.5 fold) and shock intensities (1.8 fold) than those transported using traditional PTS, rendering them more susceptible to hemolysis. This susceptibility may be associated with the direct exposure of specimens to barometric shocks. Additionally, single-pipe pneumatic logistics pipelines are typically more complex, with numerous turns and abrupt speed changes, further increasing the likelihood of hemolysis. Consequently, it is imperative to validate single-tube PTS systems prior to their implementation.

This study further investigated the impact of varying pre-transport resting times on the bias in biochemical index results between PTS and HD transmission. Blood specimens were categorized based on resting time prior to PTS transmission: Group A (immediate processing), Group B (15-min rest), and Group C (30-min rest). Our findings indicate that prolonging the resting time before single-tube PTS transmission exacerbates the bias in LDH results when compared to manual transport. The bias observed in Group A was within acceptable limits when compared to Group D, whereas the biases in Groups B and C were not. Considering that LDH is gradually released during the coagulation process, all four samples were collected, centrifuged, and detected at the same time, so as to avoid the influence of different total resting times on the test results. We hold the opinion that this discrepancy may be attributed to the gradual increase in clot volume and impact force associated with extended resting periods during PTS transport. From a physical perspective, prolonged resting may allow partial clot consolidation, increasing the effective mass and stiffness of the specimen during transport. Under rapid deceleration in single-tube PTS, this may amplify transient mechanical stress on cellular components, thereby increasing LDH release. Nonetheless, some researchers argue that clot formation may protect cells and reduce hemolysis (Koch et al., 2022), as studies indicate that procoagulant tubes are less prone to hemolysis during PTS transport compared to anticoagulation tubes (Pasqualetti et al., 2016). However, it is important to note that the researchers did not strictly control the coagulation time for procoagulant tubes, which typically ranges from 30 to 60 min. Therefore, these discrepancies may arise from uncontrolled coagulation durations in previous studies, whereas the present study isolates pre-transport resting time as an independent variable. Our study is the first to confirm the link between blood specimen standing time and result bias before single-tube PTS transport, highlighting the importance of managing placement time to ensure accurate test results. Laboratories should include this factor in their verification processes. During the installation of such a system in a laboratory setting, it is crucial to assess the system’s capacity to handle sample transport during peak blood collection periods. To minimize pre-transport waiting times, it may be necessary to increase the number of single-tube PTS pipes. This adjustment is more feasible than with traditional barrel PTS systems, as the smaller diameter of single-tube PTS pipes facilitates easier construction. We also recommend laboratories integrate blood collection pathways with the single tube PTS to decrease sample resting times. Furthermore, a modified laboratory information system should prioritize the transport of specimens containing LDH items to mitigate the impact on test results for large batch processing.

In addition, this study revealed that in the single-tube PTS transfer group, GLU concentration gradually decreased with increased resting time before transfer (Group A > Group B > Group C). It is well established that glucose is an unstable analyte when samples were stored in a silica gel procoagulant tube, which will gradually decrease over time. To mitigate its impact on the experimental outcomes, the interval from sample collection to centrifugation for the four-tube samples was standardized, as previously noted. Therefore, we assert that glycolysis is unlikely to account for the observed discrepancies. Additionally, we noted variations in other indicators across different groups. Although these variations remained within clinically acceptable limits, it would be prudent to increase the sample size to further investigate the underlying causes, if required.

Our experiment has several limitations. Our pipeline route is complex, involving both horizontal and vertical dimensions (Figure 1). Since changes in test tube orientation can impact clinical chemistry test results (Lippi et al., 2014), further investigation is needed to determine if this occurs during PTS transport. Previous studies have established a strong correlation between tube fill level and hemolysis rate (Luginbühl et al., 2024), it remains unclear whether the deceleration performance of our system is influenced by tube weight or fill level. While evaluating accelerations across multiple PTS routes helps ensure sample integrity for LDH measurements (Huang et al., 2025), a focused analysis of the acceleration profiles along our particular route—especially during the deceleration phase—would provide deeper insights and is an important avenue for further study. In the experiments conducted, the time interval between sample collection and subsequent testing for each individual was maintained consistently. However, this consistency led to varying intervals between post-PTS transport and analysis across the four groups. Drawing on previous research regarding the stability duration of each item post-sample collection (Heday et al., 2020), it is unlikely that the relatively short duration of these intervals would compromise the validity of our findings. Consequently, a comparative analysis of this timeframe across the different groups was not performed. Additionally, in our experiments, silica gel tubes were employed due to their prevalent use in the analysis of biochemical analytes. However, caution is warranted when extrapolating our findings to other types of tubes, as they may exhibit different properties that could influence stability and hemolysis rates. This study concludes that the single-tube PTS is a viable alternative to manual transport for biochemical testing, showing no significant increase in sample hemolysis. A key consideration is that extended pre-transport rest can lead to elevated LDH levels, as measured in biochemical samples collected in silica-clot activator tubes, potentially compromising result accuracy. We recommend that laboratories incorporate controls for rest time into their PTS validation protocols to mitigate this pre-analytical error.

## Data Availability

The raw data supporting the conclusions of this article will be made available by the authors, without undue reservation.
