# Supplementary material for: Navigating preanalytical challenges: a real-world study on single-tube pneumatic tube systems
Source: Front Physiol. 2026 Feb 10;17:1700252. doi: 10.3389/fphys.2026.1700252 (PMC12929093; doi:10.3389/fphys.2026.1700252)
Supplement: Supplementary file 1 [file Table1.docx]

**Supplementary Table S1 .**Timing details for the entire experimental workflow

|  | Draw to transport start(Min) | Transport duration(Sec) | | Arrival-to-centrifugation time(Min) | Centrifugation-to-analysis time(Min) | Ttotal draw-to-analysis time(Min) |
| --- | --- | --- | --- | --- | --- | --- |
|  |  | Pipeline | Secondary deceleration device |  |  |  |
| Group A | 0 | 18 | 3 | 30 | 10 | 40 |
| Group B | 15 | 18 | 3 | 15 | 10 | 40 |
| Group C | 30 | 18 | 3 | 0 | 10 | 40 |
| Group D | 0 | 600 | | 20 | 10 | 40 |

**Supplementary Table S2.** Number of removals for analytes had outliers removed

| Analytes had outliers removed | The number removed |
| --- | --- |
| LDH | 9 |
| Glu | 7 |
| 2-hydroxybutyrate dehydrogenase | 8 |
| Albumin | 3 |
| Alanine aminotransferase | 8 |
| Aspartate aminotransferase | 7 |
| Chloride | 10 |
| Creatinine | 4 |
| Gamma-glutamyltransferase | 8 |
| Magnesium | 3 |
| Inorganic phosphorous | 12 |
| K+ | 6 |
| Sodium | 5 |
| Total bilirubin | 7 |
| Total protein | 5 |
| Uric acid | 4 |
| Direct Bilirubin | 9 |
| Alkaline phosphatase | 6 |
| Urea | 2 |
| Total calcium | 4 |
| Creatine kinase | 10 |

**Supplementary Table S3.** Sensitivity analysis (All vs Removed) for LDH, GLU, and HBDH

|  | Contrast | Paired t-test p-value | Wilcoxon signed-rank p-value | Holm-adjusted paired t-test p-value | Holm-adjusted Wilcoxon signed-rank p-value | Repeated-measures ANOVA p-value |
| --- | --- | --- | --- | --- | --- | --- |
| LDH_ALL | A-D | 2.20E-03 | 2.27E-04 | 2.20E-03 | 2.27E-04 | 3.94E-04 |
| LDH_ALL | B-D | 1.84E-05 | 6.60E-06 | 5.53E-05 | 1.40E-05 |  |
| LDH_ALL | C-D | 4.21E-04 | 4.67E-06 | 8.41E-04 | 1.40E-05 |  |
| LDH_REMOVED | A-D | 5.27E-04 | 1.17E-03 | 5.27E-04 | 0.001169356 | 4.19E-15 |
| LDH_REMOVED | B-D | 7.17E-06 | 1.30E-04 | 1.43E-05 | 0.000260706 |  |
| LDH_REMOVED | C-D | 1.39E-09 | 8.69E-06 | 4.16E-09 | 2.60825E-05 |  |
| GLU_ALL | A-D | 0.006161459 | 0.000751893 | 0.012322918 | 0.001503787 | 0.0019 |
| GLU_ALL | B-D | 6.93448E-05 | 0.000231592 | 0.000208034 | 0.000694775 |  |
| GLU_ALL | C-D | 0.074642044 | 0.025631647 | 0.074642044 | 0.025631647 |  |
| GLU_REMOVED | A-D | 3.67E-05 | 0.000194419 | 7.35E-05 | 0.000388837 | 4.77E-07 |
| GLU_REMOVED | B-D | 1.13E-05 | 0.000118509 | 3.39E-05 | 0.000355527 |  |
| GLU_REMOVED | C-D | 4.05E-04 | 0.000643617 | 4.05E-04 | 0.000643617 |  |
| HBDH_ALL | A-D | 1.15E-03 | 7.93E-05 | 2.30E-03 | 7.93E-05 | 1.15E-03 |
| HBDH_ALL | B-D | 1.56E-05 | 2.05E-06 | 4.69E-05 | 6.14E-06 |  |
| HBDH_ALL | C-D | 2.68E-03 | 9.03E-06 | 2.68E-03 | 1.81E-05 |  |
| HBDH_REMOVED | A-D | 3.01E-05 | 2.74E-04 | 3.01E-05 | 2.74E-04 | 8.65E-14 |
| HBDH_REMOVED | B-D | 2.84E-07 | 1.14E-05 | 5.68E-07 | 2.58E-05 |  |
| HBDH_REMOVED | C-D | 5.26E-09 | 8.59E-06 | 1.58E-08 | 2.58E-05 |  |

**Supplementary Table S4.** Examination of biases in indices exhibiting significant differences among four transport modes

| Analyte, unit | Bias(%) | | | LoA(%) | | | 1/2TEa  (%) |
| --- | --- | --- | --- | --- | --- | --- | --- |
|  | A-D | B-D | C-D | A-D | B-D | C-D |  |
| Albumin, g/L | -0.691±1.223 | -0.008±1.22 | -0.008±1.173 | -3.1 to 1.7 | -2.4 to 2.4 | -2.3 to 2.3 | 3 |
| Alanine aminotransferase,  μkatal/L (U/L) | -2.953±6.834 | 0.564±5.083 | -2.508±9.621 | -16.4 to 10.4 | -9.4 to 10.5 | -21.4 to 16.4 | 8 |
| Gamma-glutamyltransferase,  μkatal/L (U/L) | -0.903±3.415 | 1.685±4.927 | 2.972±5.759 | -7.6 to 5.8 | -8.0 to 11.3 | -8.3 to 14.3 | 5.5 |
| Glu, mmol/L | 1.248±1.299 | 1.461±1.589 | 0.83±1.582 | -1.3 to 3.8 | -1.7 to 4.6 | -2.3 to 3.9 | 3.5 |
| LDH, μkatal/L (U/L) | 3.207±3.861 | 5.819±5.057^a^ | 10.712±6.656^a^ | -4.4 to 10.8 | -4.1 to 15.7 | -2.3 to 23.8 | 5.5 |
| Magnesium, mmol/L | -0.921±1.901 | 0.065±1.748 | 0.078±1.646 | -4.7 to 2.8 | -3.4 to 3.5 | -3.2 to 3.3 | 7.5 |
| Inorganic phosphorous, mmol/L | 0.922±2.788 | 0.668±2.24 | 0.859±1.589 | -4.5 to 6.4 | -3.7 to 5.1 | -2.3 to 4.0 | 5 |
| Sodium, mmol/L | -0.227±0.308 | -0.135±0.271 | -0.099±0.318 | -0.8 to 0.4 | -0.7 to 0.4 | -0.7 to 0.5 | 2 |
| Total bilirubin, μmol/L | -1.151±2.219 | -0.39±2.11 | -0.526±1.875 | -5.5 to 3.2 | -4.5 to 3.8 | -4.2 to 3.2 | 7.5 |
| Alkaline phosphatase, μkatal/L (U/L) | 1.142±2.662 | 0.923±2.877 | 0.732±2.592 | -4.1 to 6.4 | -4.7 to 6.6 | -4.4 to 5.8 | 9 |
| Creatine kinase, μkatal/L (U/L) | 0.791±3.295 | -1.259±3.611 | 0.452±3.934 | -5.7 to 7.3 | -8.3 to 5.8 | -7.3 to 8.2 | 7.5 |
| 1. Hydroxybutyrate dehydrogenase,   μkatal/L (U/L) | 3.446±3.34 | 6.135±4.632 | 8.083±4.959 | -3.1 to 10.0 | -2.9 to 15.2 | -1.6 to 17.8 | 15 |

Note: TEa is from the 2024 China National Health Commission Clinical Laboratory Center External Quality Evaluation Standards. Bias <1/2TEa is within the acceptable range of the laboratory. ^a^ Bias > acceptable range of the laboratory.

**Supplementary Table S5.** Repeated-measures ANOVA p-value (unadjusted) and Holm-adjusted p-value for LDH, GLU, and HBDH

| Analytes | Repeated-measures ANOVA p-value (unadjusted) | Holm-adjusted p-value for repeated-measures ANOVA across analytes |
| --- | --- | --- |
| LDH | 4.19E-15 | 8.8E-14 |
| Glu | 0.000000477 | 1.73E-12 |
| 2-hydroxybutyrate dehydrogenase | 8.65E-14 | 0.00000906 |

**Note:** p values were derived from repeated-measures ANOVA across the four transport conditions, with Holm correction applied for multiple comparisons among the 22 analytes.
